# Curcumin-loaded poly lactic-co-glycolic acid nanoparticles effects on mono-iodoacetate -induced osteoarthritis in rats 

**Published:** 2017-06-15

**Authors:** Firoozeh Niazvand, Layasadat Khorsandi, Mohammadreza Abbaspour, Mahmoud Orazizadeh, Negar Varaa, Mahtab Maghzi, Kheironesa Ahmadi

**Affiliations:** 1 *Student Research Committee, Ahvaz Jundishapur University of Medical Sciences, Ahvaz, Iran; *; 2 *Cell and Molecular Research Center, Faculty of Medicine, Ahvaz Jundishapur University of Medical Sciences, Ahvaz, Iran; *; 3 * Targeted Drug Delivery Research Center, School of Pharmacy, Mashhad University of Medical Sciences, Mashhad, Iran.*

**Keywords:** Articular cartilage, Curcumin, Mono-iodoacetate, Nanodrug, Rat

## Abstract

Curcumin has been found to be very efficacious against many different types of diseases. However, the major disadvantage associated with the use of curcumin is its low systemic bioavailability. In the present study the protective effects of curcumin-loaded poly lactic-co-glycolic acid nanoparticles (nanocurcumin) against mono-iodoacetate**-**induced osteoarthritis in rats was investigated. Mono-iodoacetate was injected into right knee joints to induce osteoarthritis. In experimental groups, 14 days after injection of mono-iodoacetate, curcumin (200 mg kg^-1^) and nanocurcumin (200 mg kg^-1^) were gavaged, respectively, for two weeks. Then the rats were sacrificed and the right knee joints were removed and fixated in 10% formalin for histological assessments. Cellularity and matrix staining were significantly increased in articular cartilage of curcumin-treated animals compared to mono-iodoacetate group (*p *< 0.01). These effects were significantly (*p *< 0.01) more in nanocurcumin-treated animals. These results suggested that administration of nanocurcumin prevented the structural changes of articular cartilage in mono-iodoacetate model of osteoarthritis in rats.

## Introduction

Osteoarthritis (OA) is a typical slow and degenerative joint disease. It affects about 80% of individuals of both sexes over the age of 60 and nearly 15% of population.^1^ The joint is a complex organ composed of different tissues including the articular cartilage, the subchondral bone, the joint capsule, the synovial membrane, synovial fluid and other soft tissue structures such as ligaments, tendons and menisci.^2^ Typically, the articular cartilage is regarded as the primary diseased tissue, and its loss of homeostasis with increased destruction and an insufficient tissue repair ultimately leads to end stage disease.^3^ There are no commercially available drugs definitely proven to modify the natural progression of OA. Non-steroidal anti-inflammatory drugs (NSAIDs) are widely prescribed for the treatment of OA pain. But the long term use of such drugs may cause side effects such as suppression of platelet aggregation, erosions and ulcerations in upper gastrointestinal tract mucosa.^4^ In fact, the current therapeutic interventions are only useful for controlling symptoms, especially pain. 

Traditionally, plants have been used to treat various health disorders.^5^ *Curcuma* *longa* is one of the most studied plants. Curcumin (Cur) is the main component of turmeric pigment of *C. longa*. Curcumin bears beneficial effects such as anti-inflammatory, antioxidant, anticancer, anti-microbial, hepatoprotective and antihyperlipidemic.^6^^-^^11^ In spite of numerous therapeutic effects, the bioavailability of Cur is low due to a relatively low intestinal absorption,^12^ rapid metabolism in liver^13^ and elimination through the gall bladder.^12^^-^^14^ It has been revealed that encapsulation of Cur in phospholipids and liposomes improves its insolubility.^15^^,^^16^ Reportedly, polymeric nanocarriers can effectively enhance therapeutic effects of Cur.^17^^,^^18^ There are reports stating nanoparticle encapsulation improves oral bioavailability of Cur up to 9-folds compared to that of free Cur.^19^ In the present study, protective effect of PLGA (poly lactic-co-glycolic acid)-encapsulated Cur (Ncur) on OA induced by mono-iodoacetate (MIA) was investigated. Intra-articular injection of MIA induces chondrocyte loss in the articular cartilage of rodent and non-rodent species. Mono-iodoacetate induces cartilage lesions with loss of proteoglycan matrix and functional joint impairment similar to human OA. ^20^

## Materials and Methods

**Animals. **In this experimental study, 40 healthy adult male Wistar rats (250 to 300 g) were used. The animals were obtained from Ahvaz Jundishapur University of Medical Sciences, Experimental Research Center, and this study was approved by the Animal Ethics Committee of Ahvaz Jundishapur University of Medical Sciences (AJUMS: 92s52) and carried out in an ethically proper way by following the guidelines provided. The animals were kept under standard laboratory conditions (12 hr-dark and 12 hr- light cycle, relative humidity of 50 ± 5% and 22 ± 3 ˚C) for at least one week before the experiment and those conditions were preserved until the end of the experiment. Animal cages were kept clean, and commercial food (pellet) and water were provided *ad libitum*.

**Preparation of NCur. **Curcumin loaded PLGA nanoparticles were prepared by solvent solid-in-oil-in-water emulsion (s/o/w) evaporation technique. Briefly, 60 mg of the PLGA were dissolved in 1 mL chloroform as an oil phase (organic solution). Free Cur (6 mg) was added to the PLGA/chloroform solution and sonicated. The emulsion was then added to a solution of ethanol and 2% PVA (1:1) and sonicated for 2 min. For evaporation (remove the organic phase) of solvent (chloroform) s/o/w emulsion was sonicated and agitated by stirrer for 5 to 6 hr. The sample was then centrifuged at 15000 *g* for 10 min and washed three times with distilled water. It was then freeze dried for 24 hr to obtain dry powder. The nanoparticles were stored at 4 ˚C for further use.^21^^,^^22^

**Characterization of NCur. **Encapsulation efficiency and particle size of the NCur were determined. The encapsulation efficiency of the nanospheres was determined analyzing the supernatant of the final emulsion once the nanospheres were removed from it by centrifugation at 15000 *g* for 15 min. For estimation of Cur present in the supernatant, the absorbance was measured spectrophotometrically at 425 nm and the amount of drug present was calculated from calibration curves of concentration versus absorbance with known standards of the drug. Encapsulation efficiency (EE) and Cur loading were calculated using formula as follow: ^21^^,^^22^

*Encapsulation efficiency (%) = (Amount of Cur in the nanoparticle / Initial amount of Cur) × 100*

*Cur loading (%) = (Amount of Cur in the nanoparticle / Total number of nanoparticle) × 100*

where,

*Amount of Cur in the nanoparticles = Total amount of Cur – free Cur*

Atomic force microscopy (AFM) method was used to determine the size and morphology of the synthesized NCur.

**Experimental** **design. **The animals were randomly divided into four groups. Saline (50 μL) was injected into the right knee joints through the infra-patellar ligament in control group. In order to induce OA, 1 mg of MIA (Sigma, St. Louis, USA) was dissolved in 50 μL saline and injected into right knee joints through the infra-patellar ligament.^4^

Two weeks after injection of MIA, 200 mg kg^-1 ^Cur and 200 mg kg^-1 ^Ncur were gavaged, respectively, for 14 consecutive days. The duration time and doses of MIA, Cur and NCur were based on previous studies.^22^^-^^25^ At the end of experiment, all rats was anesthetized by peritoneal injection of ketamine (80 mg kg^-1^; Alfasan, Woerden, Holland) and xylazine (10 mg kg^-1^; Alfasan). After inducing deep anesthesia, the rats were euthanized by gas displacement with 10% of CO_2_.^26^^,^^27^ Right knee joints of the rats were removed and fixated in formalin 10%. The fixated samples were decalcified using 5% formic acid for six days.

**Histological staining. **Paraffin-embedded blocks were cut in a 5-μm thickness and stained with Hematoxylin and Eosin (H & E) for routine histological evaluation. Safranin O (Sigma) and tolouidine blue (Sigma) staining were also used to evaluate proteoglycans and glycos-aminoglycans in the cartilage matrix. Briefly, the slides were deparaffinized, hydrated and stained by 0.10% Safranin O solution (diluted in ethanol) for 10 min. For toluidine blue staining, the dehydrated slides were placed directly into the 0.40% toluidine blue solustion (diluted in 0.10 M sodium acetate buffer) for 5 min.^28^

A modified Mankin grading was used to score cartilage change. The cartilage structure was scored on a scale of 0 - 6; where 0 : normal, 1 : irregular surface, including fissures into the radial layer, 2 : pannus, 3 : absence of superficial cartilage layers, 4 : slight disorganization (an absent cellular row and some small superficial clusrers), 5 : fissures into the calcified cartilage layer and 6 : disorganization (chaotic structure, clusters, and osteo-clastic activity). Additionally, cellular abnormalities were scored on a scale of 0 to 3; where 0 : normal, 1 : hypercellularity, including small superficial clusters, 2 : clusters and 3 : hypocellularity. The matrix staining was also scored on a scale of 0 to 4; where 4 : normal, slight reduction of staining, 3 : staining reduced in the radial layer, 2 : staining reduced in the interterritorial matrix, 1 : staining present only in the pericellular matrix and 0 : staining absent.^29^

The microscopy images were captured using the BMZ-04-DZ digital microscope (Behin Pajouhesh, Tehran, Iran). Two observers, blinded to the control and experimental groups, analyzed the sections independently.

**Statistical analysis. **The data were analyzed using one-way ANOVA followed by post hoc LSD test and were presented as the mean ± SD. A *p* value less than 0.05 was considered significant.

**Fig. 1:**
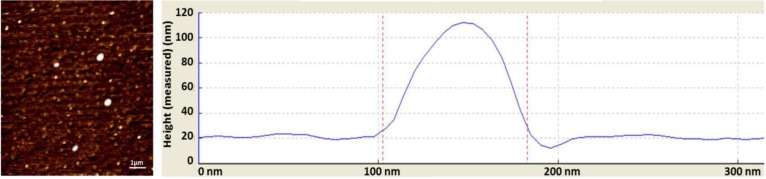
Atomic force microscopy image of nanoparticles showed distinct spherical particles in size range between 100 and 200 nm.

## Results

**Characterization of NCur. **The particle size distribution showed a range of 100 nm to 200 nm, with the mean particle size being 136 nm. The encapsulation efficiency of Cur-loaded PLGA nanospheres was 97.00 ± 0.45%. Atomic force microscopy assessments revealed the size and morphology of the synthesized NCur. As can be observed in Figure 1, the Cur-loaded PLGA showed spherical morphology and a particle size distribution almost homogeneous*,* with a mean size between 100 and 200 nm. In addition, the Cur-loaded PLGA nanospheres were completely dispersed with no aggregates in water. While, free Cur exhibited poor solubility in water.

**Histology. **The joints from non-induced OA (control group) showed smooth articular cartilage surfaces with the underneath layer of flattened chondrocytes in the tangential zone and chondrocytes were normally distributed in parallel rows transitional and radial zones of the articular cartilage. Intercellular matrix deeply and uniformly stained with Safranin O and toluidine blue.

**Fig. 2:**
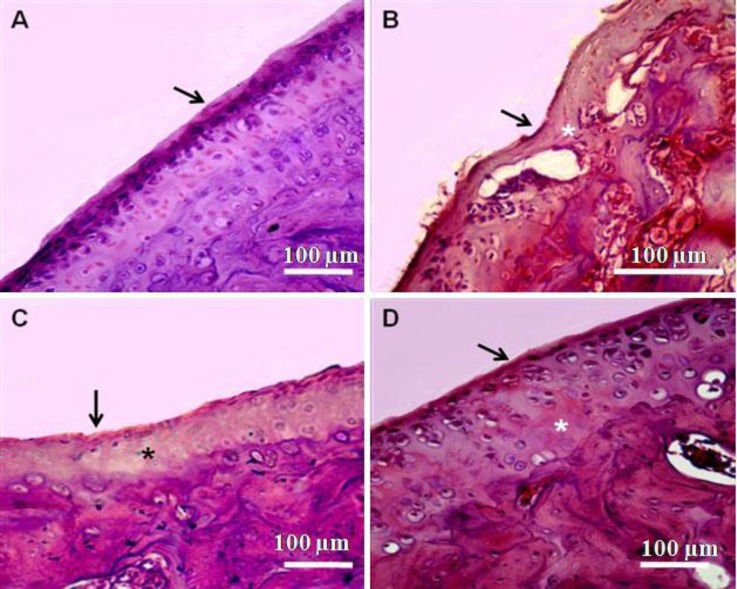
Photomicrographs of knee joints (H & E staining). A) Control group: Chondrocytes are viable across all regions of the joint surface. B) MIA treated joints: Focal loss of chondrocytes is observed in regions where tidemark integrity was breached. C) Cur + MIA group: hypocellulariy and tidemark integrity is partially improved. D) NCur + MIA group: Cellularity is increased and tidemark integrity is similar to the control. Asterisks and arrows indicate hypocellularity and tidemark, respectively

**Fig. 3:**
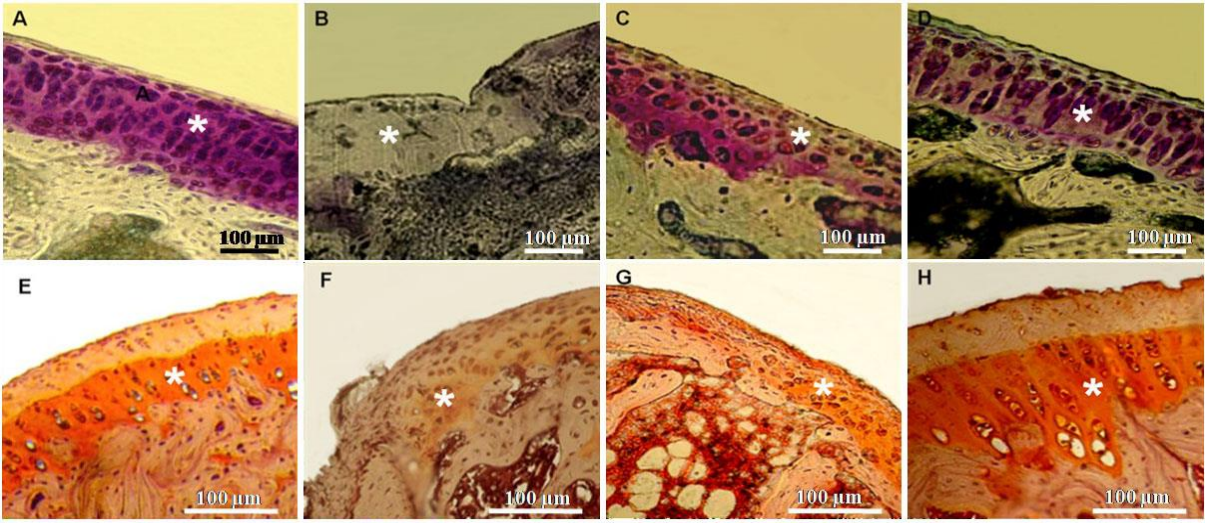
Matrix staining of knee joints. A and E) Control group: Strong staining of matrix can be seen. B and F) MIA group: Weak or negative matrix staining is observed. C and G) NCur + MIA group: Moderate matrix staining can be observed. D and H) NCur + MIA group: Strong staining of matrix similar to the control is observed. Asterisks indicate matrix staining. A-D: Toluidine blue staining, E-H: Safranin O staining

The joints of MIA-induced OA showed severe discontinuity, degeneration of the articular cartilage and disappearance of chondrocytes in the tangential, transitional and radial zones of the cartilage. The cellularity of articular cartilage was significantly decreased (*p* < 0.01). Sections stained with Safranin O or toluidine blue revealed severe reduction in their staining indicating proteoglycans loss. In Cur + MIA group, histological changes were considerably reversed. Mankin score was significantly decreased in comparison to MIA-treated animals (*p* < 0.01). Cellularity and matrix staining of articular cartilage in MIA + Cur treatment were significantly increased compared to MIA-treated joints (*p* < 0.01). Treatment with NCur could effectively improve the structural changes induced by MIA. In MIA + NCur group, cellularity and matrix staining of articular cartilage were significantly increased in comparison with MIA-treated joints. In this group, the cellularity was slightly decreased in comparison with control group (*p* > 0.01). These results are reported in Figures 2 and 3, and Table 1.

**Table 1.** Mankin scoring of knee joints in control and experimental groups

**Groups**	**Total Mankin**	**Tidemark integrity**	**Matrix staining**	**Cellularity**	**Structure**
**Control**	0.40 ± 0.03	0.00	0.16 ± 0.03	0.20 ± 0.03	0.04 ± 0.00
**Cur**	0.38 ± 0.04	0.00	0.15 ± 0.40	0.19 ± 0.05	0.04 ± 0.00
**Ncur**	0.38 ± 0.04	0.00	0.16 ± 0.80	0.18 ± 0.07	0.04 ± 0.00
**MIA**	11.6 ± 1.60*	0.80 ± 0.09*	3.86 ± 0.60*	3.20 ± 0.30*	3.70 ± 0.60*
**Cur + MIA**	5.70 ± 0.30*^†^	0.30 ± 0.07*^†^	2.60 ± 0.04*^†^	1.80 ± 0.80*^†^	1.50 ± 0.50*^†^
**NCur +MIA**	1.70 ±0.20^*†θ^	0.00	0.20 ±0.07^*†θ^	0.60 ± 0.10^*†θ^	0.90 ±0.06^*†θ^ ^θ^

Values are expressed as mean ± SD for eight rats.

* *p* < 0.001,

† *p* < 0.01,

θ *p* < 0.01,

θθ *p* < 0.001;

*, † and θ symbols, respectively, indicate comparison with control, mono-iodoacetate (MIA)-induced osteoarthritis (OA) and Cur group.

## Discussion

This study demonstrated that oral administration of Cur encapsulated in PLGA could enhance its chondro-protective effects against MIA-induced OA in rats. Zhang *et al*. revealed Cur and Ncur slow osteoarthritis progression in a post-traumatic osteoarthritis mouse model.^30^ Belcaro *et al*. demonstrated that Cur improves joint swelling, morning stiffness and walking time.^31^

The enhancement of chondro-protective effects of Cur encapsulated in PLGA may relate to increase in Cur bioavailibity and stability. In addition, Cur content in synovial fluid, extracellular matrix and chondrocytes may increase when is encapsulated in PGLA. Takahashi *et al*. showed that oral administration of a liposome encapsulated Cur to rats could significantly increase the bioavailibity of Cur compared to free Cur.^16^ The *in vivo* pharmacokinetics revealed that Cur entrapped nano-particles demonstrate at least 9-fold increase in oral bioavailability when compared to Cur administered with piperine as absorption enhancer.^19^ Cell viability studies revealed that the Cur -loaded nanospheres were able to exert a more pronounced effect on the cancer cells compared to free Cur.^21^ Cellular uptake studies in human epithelial cervical cancer cells (HeLa) exhibited enhanced intracellular fluorescence with Cur encapsulated PLGA when compared to free Cur.^22^

In the present study, histological examination using Mankin scoring showed that intra-articular injection of MIA in rat knee joint induced cartilage structural changes, matrix degradation and chondrocyte disorganization. The chronological progression of OA in this study is consistent with that reported by Naveen.^32^

Earlier studies have reported that MIA has inhibitory effect on the activity of glyceraldehydes-3 phosphate dehydrogenase in chondrocytes resulting in disruption of glycolysis, hydration of the extracellular matrix, increased extractability as well as reduced quantity and synthesis of proteoglycans, and eventually leads to cell death. Subsequently, the histological and biochemical changes occurred in the articular cartilage of the knee joint bears close resemblance to human OA.^33^^-^^35^

Our results revealed that free Cur could reverse hypocellularity in MIA-induced OA, but the increase in cellularity was more pronounced when it was encapsulated in PLGA. Reduced cellularity is a characteristic feature of OA cartilage.^36^ Actually, saving plausible number of cartilage cells in the joints articular structure is important in OA pathology and progression, because chondrocytes are the only component capable of controlling vital activities of the articular cartilage. Recent studies have shown a positive correlation between the degree of severity of OA and chondrocytes loss in both experimentally induced OA in rabbit cartilage and human OA cartilage.^37^^,^^38^

In addition to changes in the cellularity, the matrix staining was reduced in MIA- treated joints. In the present study, we used Safranin O and toluidine blue for matrix staining. Both Safranin O and toluidine blue are the most widely used stains for cartilage glycosaminoglycan and proteoglycan.^28^ Proteoglycan depletion may be secondary to cell loss due to the OA process. Extra cellular matrix proteins in cartilage are of great significance for the regulation of the cell behavior, proliferation, differentiation and morphogenesis.^39^

In the present study, Cur could enhance matrix staining, but this effect was more pronounced when encapsulated in PLGA. It is possibility that Ncur penetrates into the chondrocytes and stimulates glycosaminoglycan and proteoglycan synthesis. It has been revealed that Cur can stimulate matrix synthesis by restoring glycos-aminoglycan synthesis.^40^^-^^42^

In conclusion, NCur could effectively enhance chondro-protective effects of Cur. NCur could improve structural changes, increase cellularity and enhance matrix staining of articular cartilage in MIA-induced OA. Further experiments are needed to clarify the mechanisms of the effect of NCur on OA.
